# Picoliter‐volume inkjet printing into planar microdevice reservoirs for low‐waste, high‐capacity drug loading

**DOI:** 10.1002/btm2.10053

**Published:** 2017-02-03

**Authors:** Cade B. Fox, Cameron L. Nemeth, Rachel W. Chevalier, Joshua Cantlon, Derek B. Bogdanoff, Jeff C. Hsiao, Tejal A. Desai

**Affiliations:** ^1^ Dept. of Bioengineering and Therapeutic Sciences University of California San Francisco CA 94158; ^2^ UC Berkeley and UCSF Graduate Program in Bioengineering UCSF Mission Bay Campus San Francisco CA 94158; ^3^ Dept. of Pediatrics, Div. of Pediatric Gastroenterology, School of Medicine University of California San Francisco CA 94158; ^4^ Scienion AG Volmerstr. 7a Berlin 12489 Germany; ^5^ Center for Advanced Technology University of California San Francisco CA 94158

**Keywords:** drug delivery, inkjet printing, insulin, microdevices, nanobiotechnology, topotecan

## Abstract

Oral delivery of therapeutics is the preferred route for systemic drug administration due to ease of access and improved patient compliance. However, many therapeutics suffer from low oral bioavailability due to low pH and enzymatic conditions, poor cellular permeability, and low residence time. Microfabrication techniques have been used to create planar, asymmetric microdevices for oral drug delivery to address these limitations. The geometry of these microdevices facilitates prolonged drug exposure with unidirectional release of drug toward gastrointestinal epithelium. While these devices have significantly enhanced drug permeability in vitro and in vivo, loading drug into the micron‐scale reservoirs of the devices in a low‐waste, high‐capacity manner remains challenging. Here, we use picoliter‐volume inkjet printing to load topotecan and insulin into planar microdevices efficiently. Following a simple surface functionalization step, drug solution can be spotted into the microdevice reservoir. We show that relatively high capacities of both topotecan and insulin can be loaded into microdevices in a rapid, automated process with little to no drug waste.

## 1 Introduction

Oral drug administration is less invasive and has better patient compliance than parenteral methods making it the preferred route for both patients and physicians. However, there are significant physiological barriers present in the gastrointestinal (GI) tract, which lower the bioavailability of many drugs. The low pH of the stomach and intestinal digestive enzymes cause drug breakdown before absorption.^1^, ^2^ Furthermore, many drugs possess low solubility and/or low permeation through the epithelial mucosa of the small intestine, further reducing bioavailability of many drugs. Thus, the vast majority of biological therapeutics currently require parenteral administration due to their high molecular weights and low stability.^3^

Over the past decade, patches, hydrogels, and microparticles and nanoparticles have been explored as oral drug carriers.^4^, ^5^, ^6^ However, issues with polydispersity and drug dosing have limited the translation of these delivery methods. Previous work in our lab has demonstrated that polymeric, micron‐scale planar devices, henceforth referred to as microdevices, are capable of enhanced adhesion in the GI tract due to their high surface‐area‐to‐mass ratio and flat shape that minimizes the amount of shear stress from intestinal fluid flow.^7^ Additionally, a drug reservoir can be fabricated on one side of the disc‐shaped microdevice to further improve drug bioavailability by providing unidirectional release rather than the omnidirectional release of drug from conventional microparticles. The presence of a reservoir can protect drug payloads from the harsh microenvironment of the GI tract until release is desired. The benefits of microdevice delivery have been previously demonstrated in vivo with acyclovir where the bioavailability was observed to be 4.5‐fold higher in mice when administered in microdevices relative to a bolus dose of oral solution.^8^ These devices can be manufactured en masse in a reproducible manner by taking advantage of well‐established microfabrication techniques. The advantages of microdevices make this technology an attractive candidate for oral drug delivery of pharmaceuticals.

While microdevices are a promising technology, loading drug into the micron‐scale reservoirs of these devices in an efficient manner remains challenging. Traditional oral dosage requires large amounts of drug, which can be cost prohibitive. Previously, microdevices have been loaded by spin‐casting a drug‐hydrogel solution over the microdevices and then selectively cross‐linking the solution within device reservoirs by ultraviolet light (UV) exposure.^8^ However, drug loading efficiency is decreased due to losses during the spin‐casting step, and the volume occupied by the hydrogel reduces the drug capacity of the devices. Furthermore, UV light can damage photosensitive molecules and lead to degradation of the active compound. More recently, Fox et al. demonstrated the use of nanostraw membranes in order to passively take up drug into microdevice reservoirs via diffusion.^9^ Unfortunately, this method requires the use of concentrated drug solution that is usually discarded after loading, thus leading to waste, and drug loading capacity is limited to the product of the drug solubility and microdevice reservoir volume. Therefore, alternative loading methods need to be considered.

Inkjet printing is a technique that has been used for microarray spotting, surface functionalization, cell culturing, and drug formulation.^10^, ^11^, ^12^, ^13^ A major advantage of inkjet printing is its drop‐on‐demand mode, which allows for spotting of precise volumes of liquid onto a surface. Previously, it has been shown that small‐volume dispensing systems can be used to print polymer solutions into microscale containers.^14^, ^15^ When printing drugs, the solutions ideally would not contain polymer to maximize the free volume available for drug. These previous methods were used for tall microcontainers measuring over 250 µm in height with aspect ratios typically >1.^14^, ^15^ However, planar microdevices for oral drug delivery are typically designed with heights <10 μm and aspect ratios <0.1.^9^, ^16^ To our knowledge, this method has not been used with thin (<10 µm thickness) microdevices, which typically have reservoir volumes in the tens of pL range rather than the ≥500 pL volume reservoirs previously utilized for inkjet printing.^7^, ^14^

In this study, we demonstrate the use of picoliter‐volume inkjet printing to efficiently load planar microdevices with topotecan, a small molecule chemotherapeutic agent, and insulin, a peptide hormone. Both drug solutions were prepared in acidic solutions and were directly spotted into the reservoirs of each microdevice with high precision and accuracy in an automated fashion. We also introduce a simple surface modification step to improve the surface hydrophobicity of the microdevices, ensuring reservoir loading without overflow. We demonstrate that microdevices that can be loaded with nearly 100% of the free total volume space filled with both small molecule and biological therapeutics in an essentially zero‐waste manner.

## 2 Materials and methods

### 2.1 Materials for device fabrication and drug loading

Topotecan hydrochloride, insulin (recombinant human), hydrochloric acid (HCl), and trichloro(1H,1H,2H,2H‐perfluorooctyl)silane were purchased from Sigma‐Aldrich, USA. Poly(methyl methacrylate) (950 kDa in anisole), Shipley 1818 positive photoresist, Microposit 351 developer, and 1112A photoresist remover were purchased from MicroChem, USA. CyQUANT direct cell proliferation assay kit was purchased from Thermo Fisher Scientific, USA. Silicon wafers were purchased from Addison Engineering Inc, USA.

### 2.2 Microdevice fabrication

Microdevices with reservoirs were fabricated as previously described.^9^ Briefly, a silicon wafer was spin‐coated with poly(methyl methacrylate) (PMMA) followed by a baking step. The PMMA layer was spin‐coated with positive photoresist followed with another baking step. The wafer was then exposed to UV light through a photomask to form the device body features. The exposed wafer was developed and postbaked. The PMMA surrounding the masked region was dry etched with oxygen plasma in a Surface Technology PE1000 AC Plasma Source Reactive Ion Etcher. Remaining photoresist was removed by incubation in photoresist remover. To fabricate the reservoirs, the microdevice bodies were spin‐coated with photoresist followed by UV exposure through a second photomask with a reservoir pattern aligned over the microdevice bodies. The microdevices were then developed and dry etched, and excess photoresist was removed to form microdevices with reservoirs. The final microdevices were disc‐shaped, 200 µm in diameter and 8 µm in height. Each device contained a central reservoir 100 µm in diameter and 5.5 µm in depth, corresponding to a volume of 43.2 pL. Each 3‐inch silicon wafer contained a 4 × 4 array of 20 × 20 microdevice subarrays for a total device count of 6,400.

### 2.3 Microdevice surface modification

Prior to drug printing, the silicon wafer with fabricated microdevices was silanized with trichloro(1H,1H,2H,2H‐perfluorooctyl)silane via vapor deposition under vacuum at room temperature for 30 min. Wafers were printed the same day as silanization.

### 2.4 Drug loading into microdevices

**Figure 1:**
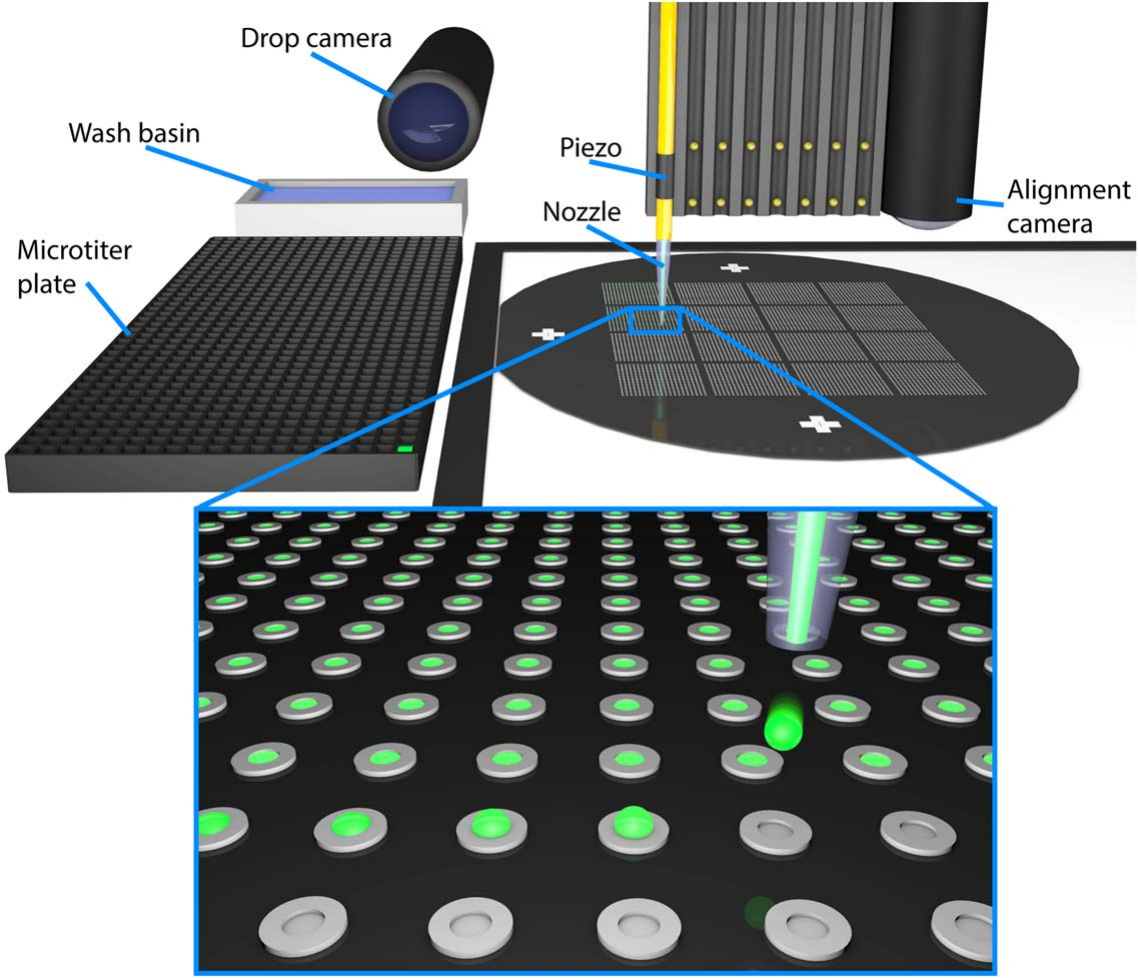
Schematic of printer configuration. Prior to each run, drug is drawn from the microtiter plate into the nozzle, and the fiduciary markers on the wafer are recognized by automated recognition of images taken by the alignment camera. Drug is then printed into microdevice reservoirs in an automated sequence. For each printing pass, the nozzle is dipped into the wash basin, and the drop camera is used to confirm successful formation of 400 pL droplets for the specified piezo settings. The printer then dispenses a single droplet into the reservoir of each microdevice on the wafer. Droplets quickly dry, and additional drug solution is printed over solidified drug in future passes. Finally, the alignment camera captures quality‐control images of all devices

Drug printing was performed using the sciFLEXARRAYER S3 (Scienion AG, Johannisthal, Germany) (Figure 1). This system is an automated picoliter drop‐on‐demand piezoelectric printing system suitable for deposition of 50‐800 pL drops at up to 1000 Hz with positional accuracy of ± 20 µm. The printer is composed of an XYZ movable head with a mounted piezo‐driven dispenser. Topotecan and insulin solutions were prepared on the day of printing at 10 mg/mL in 10 mM HCl and filtered through a 100 kDa centrifuge tube for 10 min at 5,000 relative centrifugal force. The freshly prepared drug solutions were then loaded into a microtiter plate. Prior to printing, drug solution was aspirated into the dispenser nozzle. During setup, the printer's control unit was aligned to the fiduciaries on the silicon wafer, which enabled programmable automatic dispensing. The printer is equipped with a camera to visualize drop volume, stability, and trajectory, which can be adjusted by changing the piezo voltage, pulse width, and frequency. These parameters were optimized to obtain drops with volumes of ∼400 pL. Single 400 pL drops of either drug solution were printed into device reservoirs at a rate of ∼400 devices per minute. The printing process was performed in multiple cycles to allow the solvent to completely evaporate between each cycle, preventing solution spillover.

### 2.5 Scanning electron microscopy

Samples were prepared for scanning electron microscopy (SEM) by sputter coating with 8 nm of gold followed by mounting onto carbon tape. The samples were imaged with a Carl Zeiss Ultra 55 field emission scanning electron microscope.

### 2.6 Cellular toxicity studies

To determine the cytotoxicity of silanized microdevices, samples were analyzed using a proliferation assay from the CyQUANT direct cell proliferation assay kit. Prior to use, drug‐loaded microdevices were stored desiccated at 4°C. Caco‐2 cells were grown to confluency in 12‐well tissue culture plates. All media was aspirated from wells and one of the following was added in triplicate: media containing 400 silanized and insulin‐filled microdevices, media only, or 20% dimethyl sulfoxide in media. Microdevices were scraped from the wafer with a razor and added to the media. The microdevices sunk in the media to come into direct contact with cells. The cells were then incubated at 37°C, 5% CO_2_ for 4 hrs. At the end of the incubation, cells were trypsinized and spun down to pellets via centrifugation. Trypsinizing and pelleting the cells allowed for collection of any cells that had lost adherence due to death or damage, which increased the sensitivity of the assay. The supernatant was discarded, and the cells were resuspended in PBS. Detection reagent with background suppressor was then added to each tube. The samples were incubated at 37°C for one h and then plated in a 96‐well plate in triplicate. Fluorescence of samples was measured using a spectrophotometer with cells in suspension.

### 2.7 Insulin stability assay

Microdevices were printed with 12 layers of insulin as previously outlined and stored under desiccated conditions at 4°C until stability analysis (within 1 week). Immediately before analysis, insulin was extracted from two sub‐arrays containing a total of 800 microdevices by incubating in 10 mL 0.1 N HCl at 4°C for 1 hr. Samples were then analyzed with reverse‐phase high‐pressure liquid chromatography (HPLC). For each sample, 200 μL were injected into a 50 × 2 mm Proto 200 5 μm C18 column (Higgins Analytical, USA.). A linear gradient from 30% to 35% 0.1% (vol/vol) trifluoroacetic acid (TFA) in water: 0.08% TFA in acetonitrile was applied over 10 min at a flow rate of 0.7 mL/min. Insulin and insulin degradation products were detected over time my measuring UV absorbance at 214 nm. Insulin stability was calculated as the ratio of the area of the known insulin peak to the total area of all detected peaks.

## 3 Results and discussion

Because planar microdevices can be used for oral delivery of both small molecule drugs and biological therapeutics, drug loading techniques will ideally be compatible with both small molecules and biologics in addition to being compatible with techniques for microdevice fabrication. We chose topotecan, an inhibitor of DNA enzyme topoisomerase I used as a chemotherapeutic, as a model small molecule drug.^17^, ^18^ Orally administered chemotherapy could reduce hospital admissions or visits to outpatient infusion centers for parenteral administration. However, oral formulations require higher amounts of drug to be delivered compared to the intravenous route, which can lead to higher off‐target effects and toxicity. Preparing chemotherapeutic agents in microdevice form can potentially reduce the dosage needed.

We sought to expand the utility of this platform by also examining insulin as a model biologic. Insulin is an important peptide hormone that is secreted by the beta cells in the Islets of Langerhans within the pancreas to signal for cellular glucose uptake from the bloodstream. Insulin is currently administered by multiple subcutaneous injections per day or via insulin pumps. This is not ideal due to issues such as noncompliance, cost, and tissue damage at injection sites. Oral delivery of insulin is preferable as it is a less painful administrative route and can reduce peripheral hyperinsulinemia. However, previous research investigating insulin for oral drug delivery has not been able to translate clinically due to its low stability and GI permeability.^19^, ^20^ Microdevices loaded with a high density of insulin may potentially overcome bioavailability limitations.

The shape and size of the drug delivery vehicle are important parameters to consider in oral drug delivery. We fabricated planar microdevices 200 µm in diameter and 8 µm in height. This low aspect ratio is necessary to resist shear flow from peristalsis and increase residence time. Each microdevice possesses an inner reservoir measuring 100 µm in diameter and 5.5 µm in depth. Furthermore, the reservoir is etched into only one side of the device, allowing for unidirectional drug release toward intestinal tissue and high local drug concentration at the epithelium.

The ability to load relatively large amounts of drug with minimal drug waste holds many advantages. The inkjet printer enables printing of drug directly into the reservoir and obviates the need of other methods such as spin‐casting or supercritical impregnation which can result in significant drug loss and do not guarantee uniform loading conditions.

**Figure 2:**
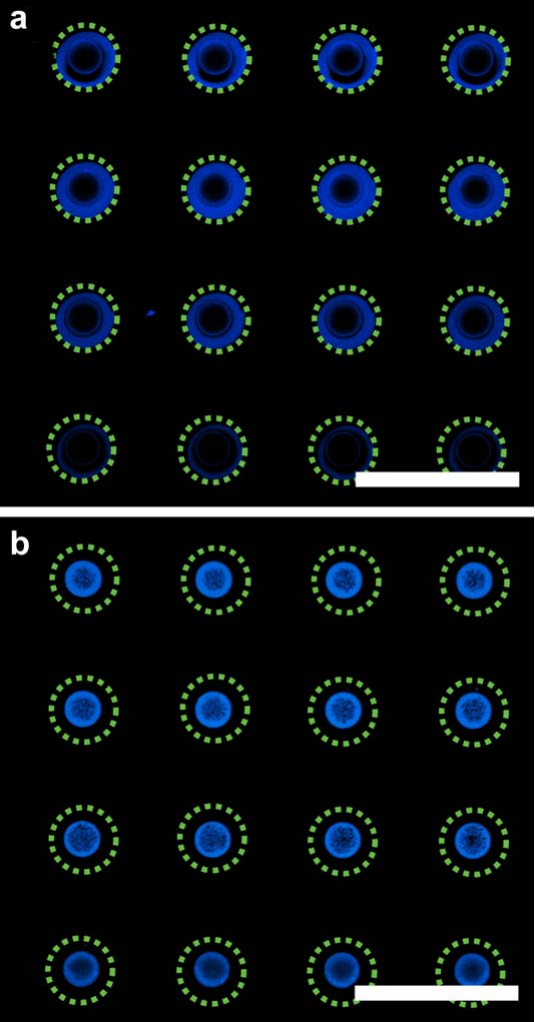
Microdevice silanization enhances drug localization into device reservoirs. **(**a) Fluorescence microscopy of devices (outlined in green dashed lines) loaded with topotecan (blue) indicates that drug spotting size was too large in nonsilanized devices, with drug being deposited both inside device reservoirs and onto the device body outside of the drug reservoirs. (b) Devices silanized with trichloro(1H,1H,2H,2H‐perfluoro‐octly)silane became more hydrophobic, providing efficient loading into device reservoirs. Scale bars are 500 µm

Surface energy is an important consideration when spotting into the shallow wells of microdevices. Before printing, the microdevices were silanized via vapor deposition of trichloro(1H,1H,2H,2H‐perfluorooctyl)silane, a silane commonly used to render surfaces hydrophobic.^21^, ^22^ The hydrophobic surface on the microdevices allowed printed drops to collect inside the reservoir (Figure 2). When loading microdevices without the silane treatment, the drops would collect outside the reservoir, which compromised loading efficiency. Following silane treatment, the drops localized into the reservoir without overflow. Silanized microdevices were used henceforth for topotecan and insulin loading.

**Figure 3:**
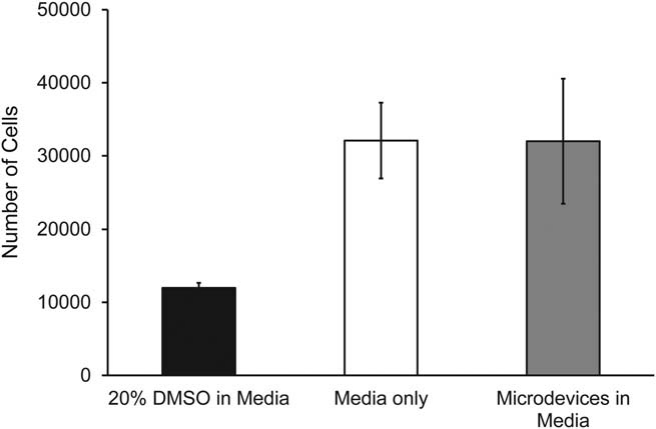
Microdevice toxicity assay. In vitro CyQUANT viability data showing that silane‐coated microdevices do not show significant toxicity to Caco‐2 intestinal epithelial cells

Drug delivery systems must also display a favorable toxicological profile in addition to demonstrating efficacy. Although we have tested microdevices in animal models before, this is the first time we have utilized silane deposition on microdevices. Silane in large quantities can be toxic to cells. However, previous studies using silanized nanoparticles did not show cell cytotoxicity suggesting the silanization process uses volumes small enough to be biocompatible.^23^ Additionally, previous work in our lab has shown these planar microdevices to have no effect on cell viability prior to drug loading.^8^ To confirm that silanized microdevices do not cause cytotoxicity and are thus biocompatible, we conducted a CyQUANT assay using Caco‐2 cells, a heterogeneous human epithelial colorectal adenocarcinoma cell line that is used as a model for the GI tract.^24^ Here, we show that silanized, drug‐loaded microdevices do not significantly decrease live cell counts compared to cells incubated in media alone (Figure 3). The DMSO group showed an expected decrease in cell viability. Assuming all microdevices lie flat on the cells, there is 38.1% coverage of the total area, which is much higher than would be expected in vivo. Either side of the microdevice could come into contact with the cells, so 50% of the microdevices have their silanized surface in direct contact with the cells. The silane is not cross‐linked to the surface, but the cytoxicity data suggest that any dissociation is not toxic at these levels. If toxicity is seen in the future, the silane could be cross‐linked. The results indicate that the silanized, drug loaded microdevices display negligible cytotoxicity, a necessary feature when translating to animal models.

The microdevices are not biodegradable on the time scale anticipated for residence in the GI tract. These devices are expected to pass completely and be excreted prior to any meaningful degradation. Additionally, the turnover time of the intestinal epithelium is 2‐3 days.^25^ Even accounting for the expected increase in residence time of the devices, they would shed with the epithelium and exit the body prior to significant degradation.

As low‐viscosity solutions (<10 cP) are most compatible with the printer, we sought to find suitable solvents for both drugs. Topotecan is soluble in aqueous solutions and is most stable at low pH values.^26^ Insulin is not soluble at neutral pH but increases solubility in acidic solutions. Thus, we dissolved topotecan and insulin at 10 mg/mL in 10 mM HCl. The measured viscosity was found to be less than 10 cP (data not shown). A silicon wafer with silanized microdevices was then placed into the printer and aligned via image recognition of the fiducial markers on the wafer before use.

Following loading of solution into the nozzle, the size of the topotecan and insulin drops was tracked by the camera and software of the printer. Before printing, 100 drops were dispensed at 200 Hz to calculate the average volume. The average drop size was determined to be ∼400 pL. Camera images taken post‐printing revealed that one 400 pL drop was sufficient to fill the entirety of the microdevice reservoir (Supporting Information Figure S1). When more than one drop was printed at a time, we observed overflow beyond the reservoir. Thus, one drop per microdevice was used during a single printing run. In order to fill the entirety of the microdevice reservoir with dry, packed drug, multiple runs were conducted with drying time between each pass.

**Figure 4:**
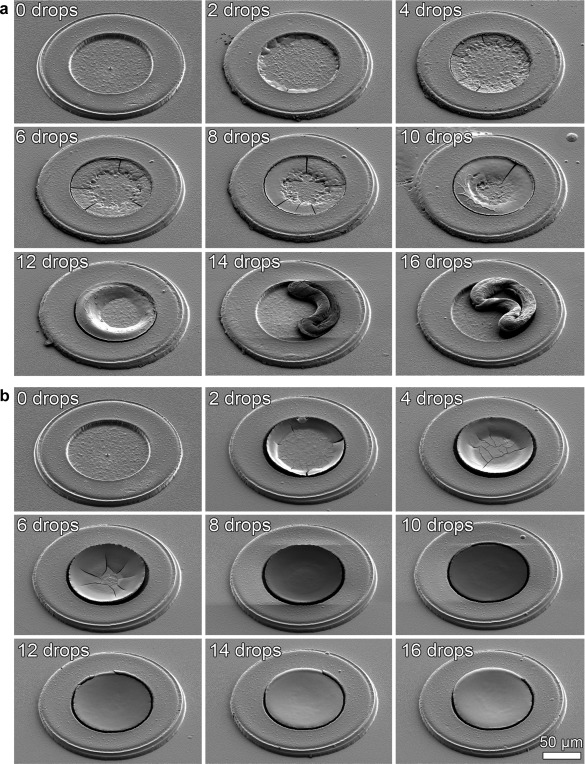
SEM images of topotecan and insulin printing. SEM images of representative microdevices loaded with increasing number of 400 pL drops of (a) 10 mg/mL topotecan and (b) 10 mg/mL insulin

To determine the optimal number of single drops that can be loaded into each microdevice, we systematically increased the number of drops up to 16 total drops per microdevice and characterized loading efficiency via SEM (Figure 4). For both topotecan and insulin, we observed gradual filling of the reservoir with increasing cycles. Imaging revealed that approximately 10 drops was the ideal number for loading topotecan while 12 drops was ideal for insulin. Topotecan loading beyond 10 drops began to overfill the microdevice reservoir until the topotecan pellet delaminated from the reservoir. Insulin loading beyond 12 drops showed overflow, but no delamination was observed. The differences in drug loading between topotecan and insulin are likely due to the two drugs' differing bulk densities.

Loading drug by printing 400 pL droplets in multiple passes allowed for significantly higher loading capacity than previous loading approaches utilizing spin casting and photolithography. For example, spin‐casting and UV‐crosslinking a 10 mg/mL insulin hydrogel solution to fill the 43.2 pL device reservoir volume would allow for loading of 0.432 ng of insulin per device. However, with inkjet printing, twelve 400 pL drops of 10 mg/mL insulin were printed into each device for a total of 48 ng insulin per device, a >100‐fold increase in loading capacity.

Topotecan is dosed based on body surface area and can be expected to be delivered in the range of 1‐4 mg/day given intravenously. Oral dosing is given for specific clinical circumstances and is threefold higher.^27^ Each microdevice contains ∼40 ng of topotecan, so administering a maximum oral dose of 4 mg of topotecan would be contained by 75,000 devices. Each 3‐inch wafer contains 6,400 microdevices, thus approximately 15 wafers would contain the equivalent dose. Insulin dosing is approximately 0.5‐1 U/kg/day divided over 3 or more subcutaneous injections.^28^ Thus, a 70‐kg person could expect to use 35‐50 U/day (1.2‐1.7 mg/day). Each microdevice contains ∼48 ng of insulin, so administering a similar amount of drug would be contained in 25,000‐37,500 microdevices or approximately 4‐6 wafers per day.

Drug stability following loading into microdevices is a concern, and biological therapeutics such as insulin are more prone to degradation than small molecule drugs due to their large molecular weight and complexity.^29^ Therefore, reverse‐phase HPLC was used to determine the stability of insulin printed into microdevices (Supporting Information Figure S2). Insulin stored in microdevices showed 21.5 ± 0.3 µg per 20 × 20 array (of the expected 19.2 µg insulin). Differences in loading may be caused by variations in droplet size during printing. Stability, calculated as the ratio of the area of the known insulin peak to the area of all peaks, was determined to be 96.0 ± 0.6% after 28 days of storage at 4°C, indicating that the printing approach has a limited impact on insulin integrity.

## 4 Conclusion

In this work, we demonstrate the use of inkjet printing to efficiently load two different drugs, topotecan and insulin, into microdevices. The advantages of the inkjet printer system lie in its high‐throughput loading efficiency, accuracy, and programmability. Spotting drug directly into the reservoir minimizes drug waste. Additionally, multiple printing and drying cycles allow for significantly higher drug loading capacity than that achieved by currently available techniques limited to loading drug at its solubility limit. Furthermore, this method does not require UV light or heat, which can damage sensitive therapeutics, and measurements of the stability of printed insulin demonstrate that biologics can be printed without significant degradation. Surface functionalization increased surface hydrophobicity, which allowed printed drug solution to localize into microdevice reservoirs and did not show significant cytotoxicity. Future studies will assess drug formulation and/or microdevice capping for tunable drug release. This inkjet printing approach could be adapted for low‐waste, high capacity loading of a number of drugs into planar microdevices for oral drug delivery.

## Supporting information

Additional Supporting Information may be found online in the supporting information tab for this article.

Supporting Information

Supporting Information
