# Supplementary figures and images for: Picoliter‐volume inkjet printing into planar microdevice reservoirs for low‐waste, high‐capacity drug loading
Source: Bioeng Transl Med. 2017 Feb 3;2(1):9–16. doi: 10.1002/btm2.10053 (PMC5426811; doi:10.1002/btm2.10053)

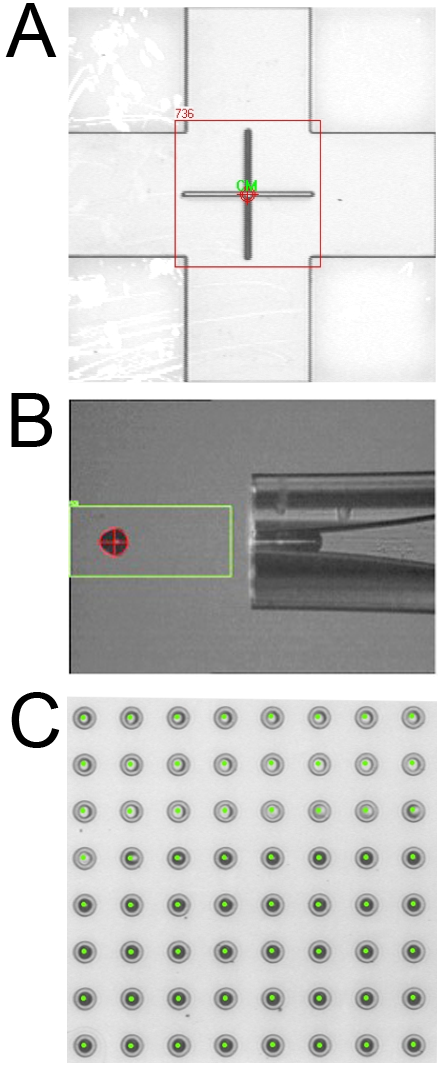

Supplement: Supplementary file 1 — Supporting Information [file BTM2-2-009-s001.tif]

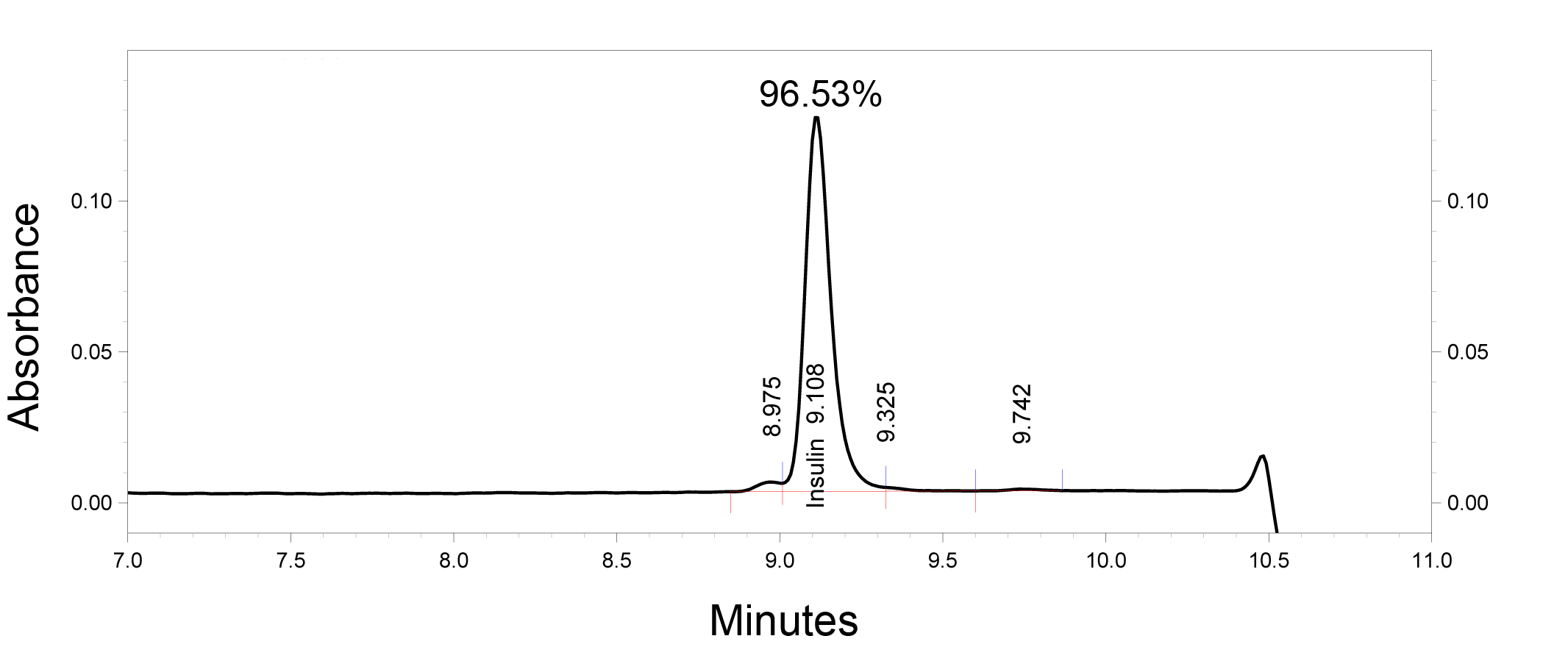

Supplement: Supplementary file 2 — Supporting Information [file BTM2-2-009-s002.tif]
